# A pilot study of the online Acceptance and Commitment Therapy Guide for Immigrant Resilience: A culturally adapted intervention for undocumented community members

**DOI:** 10.1371/journal.pdig.0001341

**Published:** 2026-04-03

**Authors:** José Manuel González Vera, Melanie M. Domenech Rodríguez, Alejandro L. Vázquez, Korena S. Klimczak, Miriam Mukasa, Guadalupe Gabriel San Miguel, Damilola Daramola, Jenifer García Mendoza, Michael E. Levin

**Affiliations:** 1 Department of Psychology, Utah State University, Logan, Utah, United States of America; 2 Department of Psychology & Neuroscience, University of Tennessee, Knoxville, Tennessee, United States of America; 3 United We Dream, Washington, D.C., United States of America; Wollo University, ETHIOPIA

## Abstract

Undocumented immigrants face chronic contextual stressors that undermine their mental health and limit their access to culturally adapted mental health interventions. This pilot implementation study evaluated the online Acceptance and Commitment Therapy (ACT) Guide for Immigrant Resilience, a seven-session self-guided program culturally adapted for young adults with liminal immigration status. Forty members of a national immigrant youth-led organization (62.5% undocumented) were given access to the ACT Guide for Immigrant Resilience and completed pre, mid, and post treatment outcome measures and a four-week follow-up. The study aimed to determine the level of engagement, acceptability/appropriateness/feasibility of the intervention, and gather preliminary effectiveness data. Participants engaged on average with 5.22 (*SD* = 2.59) of seven sessions and spent a median of 102.11 minutes with the program (Interquartile range [IQR] 25% = 54.96*,* 75% = 260.86). Implementation ratings were high, suggesting intervention acceptability, appropriateness, and feasibility. While low assessment engagement and the single-arm design limited causal inference, participant that completed post-assessment (*n* = 15) reported significant decreases in depression, anxiety, and stress, and values obstruction improvement. Reductions in depression and stress were also present at follow-up relative to baseline (*n* = 19). Qualitative feedback underscored self-compassion, values clarification, and mindfulness as being culturally relevant. Suggested improvements included clearer instructions, briefer modules, and audio options. Our findings provide preliminary support for the use of this self-guided intervention with young adults with liminal immigration status. However, randomized trials with adaptations to improve engagement are needed to further establish the efficacy of the ACT Guide for Immigrant Resilience.

## Author summary

Undocumented immigrants face many challenges that can harm their mental health, such as anti-immigrant policies, the threat of deportation, and difficulty accessing mental health care. Online mental health applications can make mental health care more accessible in this population. The ACT Guide for Immigrant Resilience is a self-paced online mental health tool designed for undocumented young adults. The focus of this study was to test the ACT Guide for Immigrant Resilience with a group of undocumented young adults. Forty members from a national immigrant youth-led organization participated in the study over the course of 12 weeks. The results suggest that the intervention helped reduce depression, anxiety, stress, and feelings of being disconnected from personal values. Based on written feedback from participants, it seemed that most believed that the content of the intervention was helpful and relatable to their experiences. These findings suggest that the intervention could be a useful and accessible mental health resource for undocumented communities.

## Introduction

Undocumented immigrants are often a forgotten group in psychological intervention research. Due to their immigration status, undocumented immigrants tend to experience chronic anxiety attributed to situational factors such as deportation, financial concerns, and lack of belongingness [1,2]. The recent COVID-19 pandemic introduced additional stressors including disruptions in family dynamics, limited access to health care, financial losses, and increased discrimination [3]. Given the unique experience of undocumented immigrants, it is important for psychologists to develop interventions that are applicable and relevant to their specific concerns.

The undocumented experience can vary based on factors such as acculturation levels, English proficiency, education, skin tone, and age of migration. Age of migration on its own can create significant differences in the experiences of undocumented immigrants. Individuals who migrated as children or adolescents, also known as 1.5 generation immigrants, tend to have greater bicultural knowledge and English proficiency than first generation immigrants, which can ease adjustment to the U.S. [4]. One of the major challenges that 1.5 generation undocumented immigrants report is struggling with navigating the stigma of not having a legal status [5]. Many recipients of Deferred Action for Childhood Arrivals (DACA) are 1.5 generation immigrations. DACA grants recipients work authorization and temporary protection from deportation in exchange for 495 U.S. dollars and personal information (e.g., address, names, birthdates of parents, place of employment) [6]. Given the additional benefits, being a recipient of DACA comes with a vastly different experience than that of other undocumented people. Simply having work authorization expands the educational and career opportunities for DACA recipients [7]. Despite its many benefits, DACA’s lack of permanence and the constant threats to end the program create a fear for its loss and a fear of having personal information used to deport recipients and their family members in the future [8].

Although there are significant differences within the undocumented experience, the common stressor is that their livelihoods are under constant threat. The last 20 years have seen an increase in anti-immigrant policies that have led to higher rates of detention and deportation [9]. Statewide policies such as Florida’s SB 1718 have unremorsefully targeted the livelihoods of this community, making it extremely difficult for many to find stable employment, access health care, and drive [10]. Increases in anti-immigrant policies worsens mental health outcomes and can lead to chronic feelings of insecurity, instability, and hypervigilance [11,12]. A survey conducted in 2020–21 by United We Dream, an immigrant youth-led organization, found an extremely high prevalence of clinical levels of self-reported anxiety (49.8%) and depressive symptoms (52.3%) in a large sample of undocumented immigrants [13]. Considering that undocumented individuals may spend decades living with the instability and uncertainty of their immigration status, it is essential for mental health clinicians to utilize treatments with long-term positive effects.

Traditional Cognitive and Behavioral Therapies (CBTs) are effective for the treatment of anxiety and depression [14–16]. Traditional CBT has also been found to be effective for treating depression and post-traumatic stress symptoms in immigrant populations [17,18]. Traditional CBT aims to reduce symptoms of psychological distress [19]. More modern, if related, psychotherapies are not as wedded to symptom reduction. For instance, Acceptance and Commitment Therapy (ACT) focuses on improvement of quality of life and does not seek to change internal experiences [20]. ACT’s therapeutic goals may be more culturally appropriate for undocumented people than traditional CBT, since the reduction of psychological distress may be difficult for this community, given the contextual factors contributing to sustained distress related to their liminal immigration status.

ACT aims to increase an individual’s ability to respond to internal experiences with psychological flexibility [21]. Psychological flexibility is defined as the ability to engage in meaningful behaviors while being aware and open to personal internal experiences (i.e., thoughts, emotions, physical sensations) [21]. Psychological flexibility has six components: acceptance, defusion, values, committed action, mindfulness, and self as context [21]. Using these six components, individuals are encouraged to engage in behaviors that improve their quality of life irrespective of what their internal experiences might be. ACT has been found to be an effective treatment for anxiety and depression [22,23].

ACT is founded on the assumption that internal experiences cannot be changed long-term and it is ineffective to expend effort on changing them [21]. For many, being undocumented is a long-term status with lasting negative mental health outcomes [11]. The circumstances they live in mean that their negative internal experiences may be a constant. ACT may help improve the quality of life for undocumented individuals by encouraging them to engage in their values and practice psychological flexibility amidst the constant instability and uncertainty they live in. Nevertheless, ACT may need to be culturally adapted to better serve undocumented immigrants. Cultural adaptations are modifications made to interventions that consider a client’s language, culture, and context [24]. Cultural adaptations can increase the effectiveness of treatments [25] and decrease the likelihood of iatrogenic effects for marginalized people [26]. Cultural adaptations of ACT have been linked to positive outcomes for Latine [27,28], Black [29], and Asian Canadian individuals [30].

The ACT Guide is an online intervention that is based on the principles of acceptance and commitment therapy [31]. The ACT Guide was developed and tested at a primarily White four-year institution in Northern Utah. The ACT Guide has been validated as a useful therapeutic substitute for internalized problems such as anxiety and depression [31–34]. Although little research has been conducted on its efficacy with marginalized groups, the principles of ACT may be applicable for the lived experiences of undocumented immigrants [35]. The ACT Guide has been culturally adapted for undocumented young adults based on feedback from community members and psychologists using the Ecological Validity Model [24,35]. The intervention adaptations included changes to improve the relevance of the material (e.g., language, examples, metaphors, content) for this population. For instance, examples involving general experiences of stress were replaced by more salient examples (i.e., “Juana is feeling anxious about her upcoming appointment with an immigration lawyer. What moves might she make to get away from this anxiety?”). Additionally, the session content and number of sessions were shortened to increase feasibility. After reviewing the feedback and consulting with the treatment developer, a total of six sessions were identified as core components of the intervention. The treatment developer also recommended having an introduction session to orient participants to the intervention. As such, the culturally adapted ACT Guide, ACT Guide for Immigrant Resilience, has seven sessions. Each session is structured with an introduction, interactive content, and a homework assignment. The introduction uses metaphors, descriptions, and images to describe a specific ACT-based skill. Afterwards, participants are provided with short practice activities related to the content. Lastly, they are provided with instructions for a homework assignment that requires practicing an ACT skill. At the beginning of each session, excluding the first session, clients are asked to reflect on their homework assignment and any challenges they encountered.

The purpose of the present study was to conduct a pilot implementation trial of the ACT Guide for Immigrant Resilience with undocumented young adults to determine whether it is an appropriate, acceptable, and feasible intervention, and to collect preliminary effectiveness data with this population. We aimed to determine (a) the level of engagement with the intervention based on the number of sessions completed, (b) effectiveness based on data from self-report measures, and (c) acceptability, appropriateness, and feasibility based on implementation outcome measures. We hypothesized that participants with higher levels of engagement would report greater improvements in mental health outcomes and psychological flexibility. Improvements in mental health outcomes and psychological flexibility were hypothesized to persist past the end of the intervention period. In addition, we hypothesized that the ACT Guide for Immigrant Resilience would be an acceptable, appropriate, and feasible treatment for undocumented young adults.

## Materials and methods

Participants included members of United We Dream (UWD), an immigrant youth-led organization. Participants were recruited with the help of a community leader within UWD. Data were collected between May 15 and September 27, 2024. We ran a power analysis for a repeated measures analysis of variance with one group and four measurements using G* Power 3.1 [36,37]. The power analysis revealed that a sample size of 24–36 would be sufficient to detect a small to medium effect size of *f* = .20 -.25 (power = .80; α = .05). Selection of effect size, power, and alpha level were based on effect sizes reported in other studies on the ACT Guide [33,38]. All participants were recruited from UWD. In order to participate in the study, individuals needed to be (a) between the ages of 18 and 32, (b) undocumented, and (c) have access to a computer with internet.

### Procedure

Data were collected using the Qualtrics survey platform. Participants were able to complete the ACT Guide and assessments during a time of day and place of their choosing. Before completing the baseline assessment, participants were presented with a consent form describing the purpose of the study and were asked whether they consented to participate before proceeding to the initial outcome survey and accessing the ACT Guide. Participants who did not consent to participate in the study were not administered surveys and did not access the ACT Guide. The design was pre-, mid-, and post-test with a four-week follow-up to examine the effects of the ACT Guide for Immigrant Resilience on participants’ wellbeing, distress, and psychological flexibility. Before accessing the intervention, all participants were instructed to complete a set of measures that assessed for overall distress, psychological inflexibility, and valued living (pre-assessment). Participants were asked about demographic information. Once participants completed the baseline surveys, they were given a unique link to access the ACT Guide for Immigrant Resilience. Participants were encouraged to complete one session per week. Participants were also recommended to wait a period of at least three to four days between sessions to allow time to practice applying the material from the intervention. However, this waiting period was not monitored to allow flexibility. Participants were sent one email at the beginning of each week containing words of encouragement and a reminder to complete at least one session that week. To provide flexibility, participants were given eight weeks to complete all seven sessions of the ACT Guide for Immigrant Resilience. After four weeks, participants were instructed to complete the same measures that were completed at the beginning (mid-assessment). At the end of eight weeks, participants were again given the same set of measures with the addition of three measures assessing the acceptability, appropriateness, and feasibility of the intervention (post-assessment). Four weeks after the conclusion of the treatment, participants were asked to complete the same measures as part of the follow-up assessment. Study procedures were approved by the Utah State University – Institutional Review Board (protocol #14051).

#### Intervention.

The ACT Guide is a 12-session, fully online, self-guided program developed by Dr. Levin and his research team at Utah State University [32]. Each session uses metaphors to assist with teaching the six core concepts of ACT. The ACT Guide for Immigrant Resilience was shortened to seven sessions due to feedback from opinion leaders asking for the ACT Guide to be shortened [35]. Session one includes a brief introduction to orient participants to the ACT Guide while also validating their experience as undocumented individuals through a self-compassion exercise. Session two defines the concept of values and helps participants identify their own values. Session three introduces the notion that experiential avoidance can worsen negative internal experiences. Session four builds on the previous session by teaching participants how to separate themselves from their thoughts. Session five teaches participants how to practice viewing their thoughts non-judgmentally by using mindfulness. Similar to session five, session six demonstrates how to respond to emotions with psychological flexibility. Session seven encourages participants to consider using their values to influence their behavior. Please see Table 1 for session content.

**Table 1:** ACT Guide sessions titles and content.

Session title	Session content
Welcome	Session one orients participants to the ACT Guide through descriptions about the intervention. Self-compassion exercises were also included to validate the participant’s experience and introduce them to the concept of psychological flexibility.
Your Values	Session two explores the meaning of values. Participants identify personal values through a value sorting activity.
Away Moves	Session three introduces away moves (i.e., experiential avoidance). Participants are gently encouraged to consider the consequences of away moves through a practice activity.
Your Mind is Like...	Session four uses metaphors to help externalize thoughts and introduce the practice of cognitive defusion.
Stepping Back	Session five includes a mindfulness activity to have participants practice defusing from thoughts.
Carrying Emotions with You	Session six uses metaphors to illustrate how emotions can impact behavior. This session also includes an activity to encourage participants to practice cognitive defusion with their emotions.
How You Want to Act	Session seven ties the previous sessions together, detailing how the practice of psychological flexibility can help improve quality of life.

### Participants

A total of 40 participants enrolled in the study. The majority (*n* = 23) were undocumented without additional legal status, while 16 were recipients of Deferred Action for Childhood Arrivals (DACA) and one was a Temporary Protective Status (TPS) recipient. Most participants (*n* = 29) were cisgender women, followed by cisgender men (*n* = 8), nonbinary individuals (*n* = 2), and one participant identifying as gender fluid. Sexual orientation was also diverse. Nearly all participants (*n* = 39) identified as Latine and were Spanish speakers. Socioeconomic backgrounds varied, with most (*n* = 25) identifying as working class and 12 as lower middle class. Regarding ability status, the majority (*n* = 32) reported no prior diagnosis of a disability, while the remaining eight had a wide range of disabilities. Educational attainment was high, with 30 participants having attended at least some college, technical, trade, or vocational school, and all participants completed high school or earned a General Education Development (GED). Employment status was split, with 22 participants holding at least one job and 14 actively seeking employment. Geographically, most participants were from the Western (*n* = 24) and Southern (*n* = 14) regions of the U.S. Lastly, about half (*n* = 22) reported having prior experience with therapy. See Table 2 for a full description of the participant demographics.

**Table 2:** Sample demographics (*N* = 40).

Characteristics	Mean/count (*SD*/%)
Immigration status	
Recipient of Deferred Action for Childhood Arrivals (DACA)	16 (40%)
Recipient of Temporary Protective Status (TPS)	1 (2.5%)
Refugee or asylum seeker	0 (0%)
Undocumented	25 (62.5%)
Immigrant visa	0 (0%)
Gender	
Agender	0 (0%)
Gender fluid	1 (2.5%)
Gender queer	0 (0%)
Gender questioning	0 (0%)
Māhū, or muxe, or two spirit	0 (0%)
Transgender man	0 (0%)
Man	8 (20%)
Transgender woman	0 (0%)
Woman	29 (72.5%)
Nonbinary	2 (5%)
Race	
Arab, Middle Eastern, or North African	0 (0%)
Asian	0 (0%)
Black or African	1 (2.5%)
Hispanic or Latine	39 (97.5%)
Native	1 (2.5%)
Pacific Islander	0 (0%)
European	1 (2.5%)
Ethnicity	
Asian	1 (2.5%)
Black	0 (0%)
Indigenous, Aboriginal, or First Nations	1 (2.5%)
Latine or Hispanic	39 (97.5%)
Middle Eastern	0 (0%)
White	5 (12.5%)
Class	
Working class	25 (62.5%)
Lower middle class	12 (30%)
Upper middle class	1 (2.5%)
Upper class	0 (0%)
I prefer not to answer	2 (5%)
Ability status	
A cognitive disability	1 (2.5%)
A learning disability	1 (2.5%)
A long-term medical illness	1 (2.5%)
A long-term medical health condition	3 (7.5%)
A mobility impairment or physical disability	2 (5%)
A sensory impairment (e.g., vision, hearing)	1 (2.5%)
A sensory processing or integration disorder	0 (0%)
A temporary impairment resulting from illness or injury	0 (0%)
A disability or impairment not listed here	1 (2.5%)
Did not identify with a disability or impairment	32 (80%)
Prior mental health diagnosis	
Yes	8 (20%)
No	0 (0%)
Education	
Elementary school	0 (0%)
Middle school	0 (0%)
Some high school	0 (0%)
High school or GED	10 (25%)
Some college, technical, trade or vocational school	11 (27.5%)
College, technical, trade or vocational school	17 (42.5%)
Graduate school	2 (5%)
Employment	
Not employed, and not looking for work	3 (7.5%)
Not employed but looking for work	14 (35%)
Retired	0 (0%)
Have one job	22 (55%)
Have two jobs	0 (0%)
Have three or more jobs	1 (2.5%)
Language	
Korean	1 (2.5%)
Russian	1 (2.5%)
Spanish	39 (97.5%)
Other	1 (2.5%)
Region	
Midwest	4 (10%)
Northeast	1 (2.5%)
South	11 (27.5%)
West	24 (60%)
United States territory	0 (0%)
Sexual orientation	
Asexual or aromantic	0 (0%)
Bisexual	8 (20%)
Demisexual	1 (2.5%)
Gay	3 (7.5%)
Lesbian	3 (7.5%)
Pansexual	3 (7.5%)
Queer	0 (0%)
Questioning	1 (2.5%)
Sexually fluid	1 (2.5%)
Straight or heterosexual	23 (57.5%)
Prior psychotherapy	
Yes	22 (55%)
No	18 (45%)

### Measures

#### Demographics.

Participant demographic information were collected to provide a thorough and accurate description of the sample. Participants were asked about age, education, gender, sexual orientation, race, ethnicity, socioeconomic status, disability, language, employment status, region they lived in, access to the internet, and immigration status. Questions and answer choices were formulated based on the article by Hughes and colleagues [39]. Participants were encouraged to fill out all questions but were allowed to skip questions that felt uncomfortable to answer. The comfort of these participants was important given the constant lack of safety many of them may live with. For screening purposes, participants were required to answer questions about age, access to the internet, and immigration status. All participants were given an identification code to ensure their data remained anonymous.

#### Open-ended questions.

Participants answered three open-ended questions at the end of each session describing what they liked best about the session, what was the most important thing they learned, and what they liked least about the session.

#### Psychological inflexibility.

The Acceptance and Action Questionnaire (AAQ-II) is a seven-item inventory that measures psychological inflexibility with a primary focus on experiential avoidance [40]. The AAQ-II is rated on a seven-point Likert scale from 1 (*never true*) to 7 (*always true*), with higher scores indicating greater levels of psychological inflexibility. The AAQ-II has excellent internal consistency (α = .84) and good convergent validity [40]. The AAQ-II has been used to measure psychological inflexibility in samples with Asian, Black, and Latine individuals [41–43]. Internal consistency within the present sample was excellent for the AAQ-II (α = .91).

#### Values.

The Valuing Questionnaire (VQ) is a 10-item measure of engagement in values broken up into two subscales: Progress and Obstruction [44]. Items are rated on a six-point Likert scale, ranging from 0 (*not at all true*) to 5 (*completely true*). Progress assesses committed action towards values while Obstruction measures whether internal experiences interfere with valued living. Higher scores in Progress and lower scores in Obstruction suggest that individuals are living a life aligned with their values. The VQ has demonstrated good convergent validity and internal consistency with populations from the United States (α = .81 for Progress and α = .79 for Obstruction) [44] and Colombia (α = .85 for Progress and α = .84 for Obstruction) [45]. Internal consistency within the present sample was excellent for the Progress (α = .75) and Obstruction (α = .87).

#### Mental health problems.

The Depression/Anxiety/Stress Scale-21 (DASS-21) is a 21-item measure of depression, anxiety, and overall stress [46]. Items are rated on a four-point Likert scale from 0 (*did not apply to me at all*) to 3 (*applied to me most of the time*). Higher scores indicate greater levels of psychological distress. The DASS-21 has been used consistently in research involving online versions of ACT due to its good internal consistency (α = .94 -.95) and its ability to detect clinically significant change [34,38]. Research has found the DASS-21 to be a useful measure of depression, anxiety, and stress for Latino, Asian, and Black individuals [47]. However, varying convergent validity across race/ethnicity suggests using caution when comparing scores cross-culturally. Internal consistency within the present sample was acceptable for the depression (α = .88), anxiety (α = .83), and stress scales (α = .77).

#### Well-being.

The Mental Health Continuum Short Form (MHC-SF) is a 14-item measure of positive mental health [48]. The measure has three subscales related to psychological, social, and emotional well-being. Responses range from 0 (*never*) to 5 (*always*), with higher scores indicating greater levels of mental health. The MHC-SF has been shown to have good internal consistency (α = .81 -.95) and convergent validity [49–52]. When used in Asian, Latin American, African, and Western countries, minimal invariance has been found, supporting its applicability and relevance across cultures [53]. Internal consistency within the present sample was acceptable for the psychological (α = .78), social (α = .77), and emotional well-being scales (α = .92).

#### Implementation outcome measures.

Acceptability, appropriateness, and feasibility are essential components to intervention implementation research (Weiner et al., 2017). These three concepts help ensure that an intervention is applicable, suitable, and sustainable for specific populations. Acceptability was measured using the Acceptability of Intervention Measure (AIM). Appropriateness was assessed through the Intervention Appropriateness Measure (IAM). Lastly, feasibility was examined with the Feasibility of Intervention Measure (FIM). The AIM, IAM, and FIM contain four items each with responses ranging from 0 (*completely disagree*) to 5 (*completely agree*). All three measures have good internal consistency (α = .85 -.91) and test-retest reliability (.73 -.88; Weiner et al., 2017). Internal consistency within the present sample was acceptable for the AIM (α = .93), IAM (α = .88), and FIM (α = .85).

#### Intervention and assessment engagement.

A secondary outcome of the study is engagement with the intervention and assessments. Engagement with the intervention was defined as the number of sessions participants completed by the end of week 8 and the time spent on the intervention in minutes. Engagement with the assessment was assessed as the number of participants who provided information for each assessment point.

### Qualitative analysis

Thematic analysis was used to categorize the data collected from the open-ended questions. The data was coded by two researchers individually for session one using inductive open coding (JMGV, DD). Inductive open coding is used as an early step in the analysis process to help researchers understand the data [54]. Afterwards, coders reviewed their individual codes and compared them to one another. The coders found an 82% similarity rate between their codes. Given the high similarity between their codes, it was deemed appropriate to split the open coding workload. Thus, one coder examined the data for the first half of participants, and the second coder examined the data for the second half of participants. Once all the data had been coded, the primary researcher (JMGV) used those codes to identify patterns and create a shortened list of codes. The primary researcher then used this shortened list to recode the data.

### Quantitative analytic plan

Descriptive statistics were used to assess engagement with the intervention (i.e., number of sessions completed, time) and assessment (i.e., number of assessment points completed). To examine whether attrition introduced bias, bivariate Kruskal-Wallis Rank Sum Test and Kendall’s Tau correlations were conducted between baseline outcome measures and missingness indicators at each assessment point. These analyses revealed that there was potential for selection bias at mid-assessment. Additionally, several outcome measures had high skew and kurtosis. Thus, nonparametric Wilcoxon Signed-Rank Tests were used to examine differences between baseline scores and outcomes at other assessment points. Pairwise comparisons were restricted to participants with complete data at both assessment points to minimize bias from selective attrition. This analytic strategy allowed for the evaluation of intervention effects while accounting for missing data and non-normality. We used the Reliable Change Index (RCI) as a post hoc test to determine whether significant changes between assessment points were beyond measurement error. The RCI is a standardized statistic that determines whether an individual’s score change between assessment points exceeds what would be expected due to measurement error alone (± 1.96) [55].

## Results

### Intervention engagement

**Fig 1:**
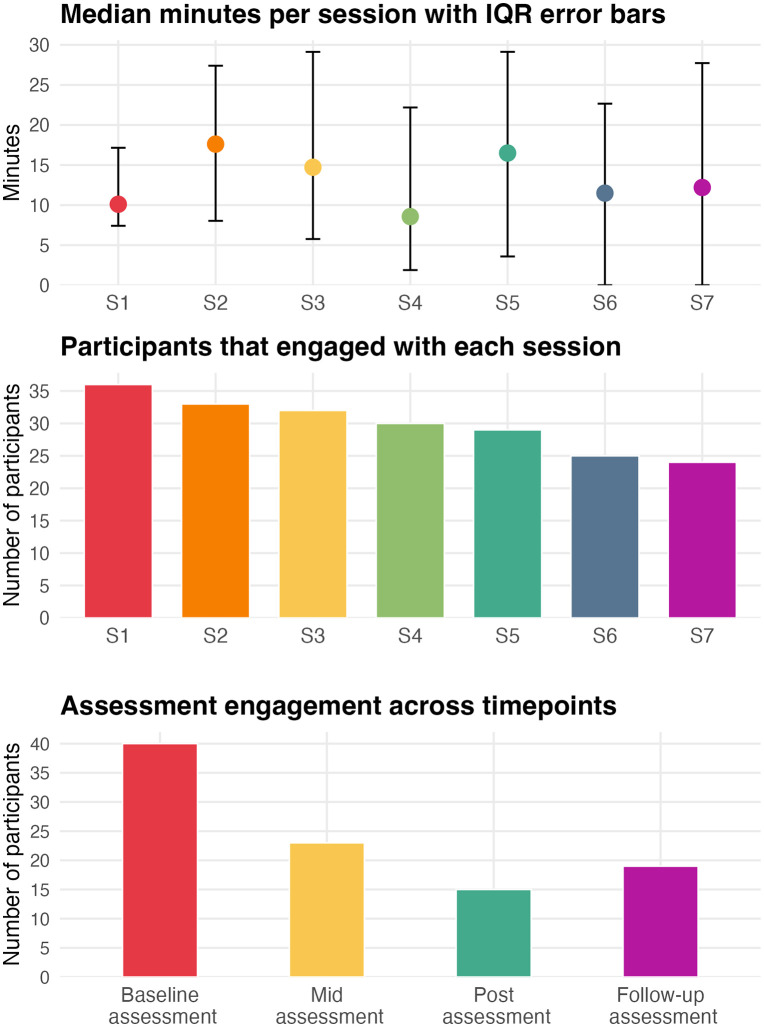
Intervention and assessment engagement data. IQR = Interquartile range. S = Session.

On average, participants engaged with 5.22 (*SD* = 2.59) sessions and spent a median of 102.11 minutes (Interquartile ran*ge [IQR] 25%* = 54.96*,* 75% = 260.86) or roughly 1.70 hours on the intervention. Participants spent the least amount of time on session four (Median = 8.57; *IQR 25%*= 1.89*,* 75% = 22.19) and spent the most time on session two (Median = 17.61; *IQR 25%*= 8.03*,* 75% = 27.40). See Fig 1 for engagement indicators. Additionally, the number of sessions completed was not significantly associated with any baseline study measures, suggesting that engagement in the intervention did not significantly differ between respondents based on their initial scores. See S1 Fig for bivariate Kendall’s Tau correlations. Lastly, measures of implementation assessing acceptability (*n* = 15; *M* = 4.82; *SD* = 0.35), appropriateness (*n* = 15; *M* = 4.52; *SD* = 0.47), and feasibility (*n* = 15; *M* = 4.73; *SD* = 0.42) were generally high among participants who completed the post-assessment. This suggests that the intervention was perceived positively by respondents.

### Assessment engagement

Participants completed 2.42 (*SD* = 1.13) assessment points on average, which included baseline (*n* = 40; 100%), mid (*n* = 23; 57.5%), post (*n* = 15; 37.5%), and follow-up (*n* = 19; 47.5%). An indicator representing whether participants were missing data on at least one variable at each time point was used to determine whether the propensity for missingness on assessment points was associated with baseline scores on outcome measures. Participants with complete data for the mid-assessment had significantly lower baseline depression, anxiety, and psychological inflexibility relative to those who had incomplete data. Thus, differences between these time points may be biased by attrition of participants who presented with higher severity at baseline. Participants’ baseline scores were not associated with the propensity for missingness for post- and follow-up assessment points, which suggests that differences between these time points and the baseline on study outcomes did not appear to be associated with a selection bias (e.g., higher or lower severity cases being retained). See S1 Table for assessment completion by baseline outcomes. The number of assessment points completed was also not significantly associated with any of the baseline measures, which suggests that the number of assessments completed by participants was not associated with baseline symptomatology, wellbeing, and indicators of psychological flexibility (See S1 Fig. for bivariate correlations).

### Intervention effectiveness

At baseline, participants reported higher levels of stress (*M* = 2.42; *SD* = .65) than depression (*M* = 2.09; *SD* = .71) and anxiety (*M* = 1.99; *SD* = .65). However, these scores indicated that participants experienced relatively low levels of stress, depression, and anxiety. Additionally, participants reported experiencing higher levels of emotional wellbeing (*M* = 3.89; *SD* = 1.21) and psychological wellbeing (*M* = 3.87; *SD* = .97) than social wellbeing (*M* = 2.77; *SD* = .97). Participants reported experiencing high levels of psychological inflexibility as suggested by their scores on the AAQ-II (*M* = 3.94; *SD* = 1.46). Their scores on the VQ-progression subscale (*M* = 4.56; *SD* = 1.06) and VQ-obstruction subscale (*M* = 3.98; *SD* = 1.37) suggested that they had a moderate alignment with their values and low levels of cognitive fusion and avoidance.

As responses on several outcome measures had high skew and kurtosis, we used a nonparametric test, Wilcoxon Signed-Rank Test to examine differences between timepoints. See S1 Table for outcome descriptive statistics. To account for data missingness and prevent bias from selective attrition, comparisons for each time point were made based on participants who had available data for each pair of assessment points. This approach helped mitigate the risk of biasing our findings due to the loss of participants with higher or lower symptom severity at baseline. From baseline- to mid-assessment, significant changes were observed only for stress. Specifically, those who completed the mid-assessment had significantly lower depression symptoms (*r* = .68) and stress relative to their baseline score with a large effect size (*r* = .62). Participants who completed the post-assessment had significantly lower levels of depression (*r* = .71), anxiety (*r* = .62), stress (*r* = .80), and values obstruction (*r* = .74) relative to their baseline scores with large effects. Participants who completed the follow-up assessment had significantly lower levels of depression (*r* = .51) and stress (*r* = .63) relative to their baseline scores with large effect sizes. In summary, the intervention appeared to be most effective in reducing depression symptoms and stress levels, with improvements from baseline among those who completed the follow-up assessment. See Table 3 for study effectiveness outcomes.

**Table 3:** Differences between time points.

Outcomes	Pre	Mid	*p*	*r*	Pre	Post	*p*	*r*	Pre	Follow-up	*p*	*r*
	*n* = 21	*n* = 21			*n* = 15	*n* = 15			*n* = 19	*n* = 19		
DASS Dep	1.84 (0.62)	1.60 (0.56)	.024	.68	2.11 (0.72)	1.50 (0.45)	.008	.71	2.22 (0.67)	1.68 (0.66)	.004	.54
DASS Anx	1.79 (0.65)	1.50 (0.38)			1.88 (0.61)	1.45 (0.40)	.037	.62	1.99 (0.57)	1.68 (0.56)		
DASS Str	2.25 (0.64)	1.85 (0.45)	.031	.62	2.43 (0.68)	1.79 (0.38)	.012	.80	2.57 (0.65)	2.04 (0.64)	.015	.63
MHC Emo	3.97 (1.14)	4.13 (1.12)			3.67 (1.24)	4.29 (1.03)			3.60 (1.13)	4.11 (0.87)		
MHC Soc	2.90 (0.91)	3.09 (1.03)			2.68 (1.01)	3.57 (1.31)			2.55 (0.87)	3.18 (1.21)		
MHC Psy	3.98 (1.01)	3.70 (1.07)			3.69 (1.05)	4.02 (1.14)			3.65 (0.92)	3.87 (0.91)		
AAQ Inflex	3.50 (1.22)	3.39 (1.12)			3.79 (1.18)	3.01 (0.92)			3.99 (1.27)	3.23 (1.38)		
VQ Pro	4.64 (0.97)	4.72 (0.94)			4.36 (0.96)	4.88 (0.94)			4.21 (0.87)	4.68 (1.04)		
VQ Obs	3.75 (1.08)	3.24 (0.82)			4.08 (1.25)	3.09 (1.04)	.038	.74	4.19 (1.33)	3.43 (1.40)		

*p* = *p* value; *r* = effect size; DASS = Depression/Anxiety/Stress Scale; MHC Emo= Mental Health Continuum - Emotional; MHC Soc = Mental Health Continuum - Social; MHC Psy = Mental Health Continuum - Psychological; AAQ Inflex = Acceptance and Action Questionnaire; VQ Pro = Valuing Questionnaire - Progress; VQ Obs = Valuing Questionnaire - Obstruction.

### Post hoc analyses

Improvements and deteriorations were observed across each time interval (see S2–S4 Figs). However, since the study did not involve a control group, there is not enough evidence to infer causation. Between pre and mid assessments, reliable improvement beyond the threshold of chance or measurement error was observed in three participants’ depression scores and five participants’ stress scores out of 21. Additionally, one participant exhibited a reliable deterioration in depression during this period. From pre and post assessments, 15 participants demonstrated reliable improvement, distributed across the following outcomes: depression (*n* = 3), anxiety (*n* = 4), stress (*n* = 4), and values obstruction (*n* = 6) scores. Reliable deterioration was observed for one participant’s values obstruction score during this interval. Between pre and follow-up assessments, seven participants experienced reliable improvement in depression scores and three in anxiety scores out of 19. Two participants’ depression scores reliably deteriorated from pre to follow-up assessment.

### Open-ended questions

Overall, there was significantly more positive feedback than negative. The questions that asked about what participants liked best and what they learned produced similar feedback for all sessions, suggesting that the two questions might have been too similar. As such, the feedback from both questions were combined into one theme: positive feedback. Activities and relatable content were identified as subthemes. The majority of participants did not offer critical feedback when asked about what they liked least, and many mentioned that there were no aspects of the sessions they disliked. Feedback about what they liked least was thematically categorized as constructive feedback. Session one received the most feedback from participants (*n* = 33) and session seven received the least feedback (*n* = 17).

#### Positive feedback: Activities.

Participants most frequently provided positive feedback about the experiential activities embedded within each session. In session one, participants appreciated that both self-compassion activities involved an acknowledgment of common systemic barriers that undocumented people face. One participant expressed: “I really liked how there was an honest acknowledgment of our everyday struggles.” In session two, participants noted that they benefited from the value sorting activity and the values vs. rules activity. They explained that these activities helped them reflect on their values and differentiate them from rules. One participant reported that this session helped them “stop and think about what I do and the reasoning behind it.” In session four, participants reported that they enjoyed the leaning close to the screen activity. They explained that the act of leaning close to the screen and then leaning away helped them learn how to defuse themselves from their thoughts. One participant stated that this activity helped them learn to “let thoughts pass. It’s okay to let myself feel the effects of nervousness as long as it isn’t keeping me from doing what I want to.” In session five, participants mentioned that they benefited from the guided mindfulness exercises (i.e., leaves on a stream, labeling your experiences). They noted that these exercises provided ways to defuse from their thoughts by viewing them objectively. One participant mentioned that “I liked the focus on separating one from our thoughts. I can often spiral with my thoughts and being able to identify when I have glasses on and label my thoughts, I am able to calm down and let things pass by.” Others also reported that they enjoyed the audio component of the activities: “I really enjoyed listening to the audio exercise where I got to practice putting thoughts on a leave…it was fun using my imagination for the exercise.” In session seven, participants mentioned that they enjoyed the letter to self activity: “I LOVED LOVED LOVED writing a letter to my future self…it made me reflect a lot and notice all the accomplishments I have achieved thus far.” They explained that this activity helped them reflect on their values and actions.

#### Positive feedback: Relatable content.

Participants also emphasized the importance of the intervention’s relatable content and examples, noting that these elements fostered validation, normalization, and a sense of shared experience. Several participants described feeling less isolated after engaging with the material, with one stating, “I felt like my emotions and experiences were not so unique for the first time.” Content that explicitly reflected the lived realities of undocumented young adults, such as metaphors, examples, and descriptions, was described as particularly impactful. In session three, participants appreciated that the hungry tiger and brick house metaphors described the consequences of experiential avoidance in contextually appropriate ways, with one participant disclosing that: “what I liked best about this session was learning about what away moves are and what they look like in the lives of undocumented individuals, like me.” In session six, participants stated that they liked the passengers on the bus metaphor and the getting off your buts activity. They commented that this content provided them with tools to co-exist with their emotions. One participant stated, “the most important thing I learned was to go forward with the action I want to do even though I have negative feelings about it.” Some participants expressed that session seven made them feel more in control of their lives: “The most important thing I learned from this session is that while you might not always control the outcomes of your actions, you have full control over how you act.” This was an important finding given that this community often feels a lack of control in their lives.

#### Constructive criticism.

Although most participants stated that they had no critiques of the intervention, there were some notable suggestions from participants. Namely, a few participants reported that the content in sessions one, two, three, four, and six were experienced as dense and would benefit from greater clarity and brevity. Other participants asked for sessions to include more examples. Some participants also experienced difficulties accessing the audio format of the guided mindfulness exercises in session five. There was some conflicting feedback with a few participants requesting more guided exercises, while others preferred less. Lastly, some participants reported experiencing difficulty applying the values-based content to their life. Please see the S3 Table for full results.

## Discussion

This study builds on the existing literature examining the efficacy of ACT-based online interventions, particularly with marginalized populations. Previous research has demonstrated the utility of online interventions for undocumented immigrants [56]. However, there remains a significant gap in understanding the effectiveness of ACT-based online interventions tailored specifically for this population**.** To address this gap, the present study evaluated the feasibility, acceptability, appropriateness, and preliminary effectiveness of the culturally adapted ACT Guide for Immigrant Resilience. Our preliminary findings support the feasibility, acceptability, and appropriateness of this intervention with undocumented young adults. Consistent with prior research on ACT-based online interventions, this study found that participants reported significant reductions in levels of depression, anxiety, and stress between assessment points [38,57]. This pattern is consistent with previous findings indicating that ACT-based interventions may foster long-term improvement in these symptoms [33]. Participants also reported a significant decrease in values obstruction after utilizing the intervention, suggesting that participants may have experienced fewer disruptions to value-based living. Despite these promising preliminary findings on intervention effectiveness, it should be noted that the present study was a single-arm implementation trial. Thus, it is difficult to determine whether improvements in study outcomes were attributed to engagement with the intervention or other factors. Future randomized controlled trials are needed to confirm the effectiveness of the ACT Guide for Immigrant Resilience.

While pilot data for the ACT Guide for Immigrant Resilience appear to provide initial evidence of effectiveness among undocumented young adults, RCI analyses suggest that reliable improvements between assessment points were only reported beyond change/measurement error for a subset of participants in the sample. Further, a small number of participants reported reliably that their depression and values obstruction scores worsened [1–2], which may necessitate additional study to determine what may have contributed to potential small iatrogenic effects on some outcomes within this population. However, given the lack of a control group and randomization procedure, it is not possible to establish whether this reported deterioration is potentially a result of participating in the intervention or related to outside factors. Future research should examine the efficacy of the ACT Guide for Immigrant Resilience in a larger sample of undocumented young adults to further evaluate this emerging intervention.

Additionally, low levels of engagement in study assessments presented a challenge in fully capturing the potential effectiveness of the intervention. Low engagement rates are common in online-based interventions [33,58,59]. The reasons for participants’ low engagement in assessments for the present study remain unclear, though one possible explanation might be that, unlike the intervention, the content of the questionnaires was not tailored for the undocumented experience. An increase in contextual relevance of the questionnaires has been found to have a positive impact on attrition rates [60]. It is also possible that low assessment engagement may have also resulted from the measurement burden of completing four survey batteries.

Engagement with the intervention was encouraging, with about 50% of participants completing the entire intervention and approximately 75% completing at least half. These engagement rates are comparable to those observed in similar digital mental health interventions [33]. This finding suggests that digital interventions may be feasible for undocumented young adults. Open-ended feedback further underscored participant perceptions of the intervention’s reliability and engagement. Specifically, participants found the metaphors used in the program informative and applicable, even those that were not modified during the cultural adaptation process. This finding is particularly significant given that metaphors play a central role in ACT [61] and are an important part of the cultural adaptation process [25]. Positive feedback towards the culturally adapted examples further supports the applicability and relatability of the intervention for this population. These findings highlight the importance of cultural adaptations, supporting the notion that even minor cultural adaptations can enhance the relevance and impact of therapeutic interventions [62]. Participants also reported enjoying the interactive activities, reinforcing the importance of active engagement in fostering psychological wellbeing. They particularly enjoyed the mindfulness and values-based exercises, which may suggest that mindfulness and values are salient concepts for this population. This finding aligns well with previous studies that have supported the integration of mindfulness [63] and values-based approaches in interventions for immigrant populations [64].

While the quantitative data may suggest some limitations in the intervention’s overall potential effects, the qualitative data provided valuable insights into participant’s experiences with the intervention. Many participants reported that the intervention was helpful and applicable to their experience, highlighting the importance of integrating qualitative methods in intervention research. A mixed-methods approach can provide a more nuanced understanding of intervention outcomes, capturing subjective experiences that may otherwise go unnoticed. Future intervention research should incorporate qualitative methods to help refine interventions based on the subjective experiences of participants.

This study provides preliminary support for the feasibility, acceptability, appropriateness, and effectiveness of the ACT Guide for Immigrant Resilience as an intervention for undocumented young adults. Although challenges such as participant engagement with assessments may point to areas for improvement, the intervention’s demonstrated potential benefits for reducing depression, stress, and values-based living highlight its potential as a meaningful mental health resource. Future research should focus on strategies to enhance engagement, explore long-term effects, and increase cultural adaptations to maximize the impact of the intervention. Consistent with the iterative process of culturally adapting interventions*,* information resulting from this implementation trial may also help inform and guide improvement for the ACT Guide for Immigrant Resilience or other digital interventions for this population [65].

### Limitations and future directions

It is important to recognize that this was a small, exploratory implementation trial. Similar to prior digital intervention studies, the present study had challenges associated with participant engagement in the intervention and outcome assessments [33,58,59]. The low assessment completion rate made it difficult to assess the intervention’s effectiveness. It is possible that participant measurement burden was too high (i.e., complete sessions, answer open-ended questions after each session, fill out four surveys) and it may be beneficial to decrease participant measurement burden in future studies. Additionally, two out of the three open-ended questions provided similar data. Some participants even copied their answer to the first question and used it as their response for the second question, suggesting that those two questions were likely too similar. Future studies should either include more distinct questions or simply use fewer open-ended questions. Propensity for missingness was examined against intervention engagement and baseline assessment data to identify potential biases in our analyses. These analyses suggested that there was not a selection bias associated with study engagement metrics and the intervention effectiveness outcomes with the exception of three midpoint outcomes (i.e., depression, anxiety, psychological flexibility). As a result, findings related to these effectiveness outcomes from the midpoint assessment should be interpreted with caution. Future research should utilize evidence-based methods of improving intervention and assessment engagement such as additional compensation for high assessment engagement and supplemental coaching [58]. It is also possible that analyses were underpowered to find differences between timepoints due to low engagement with outcome assessments. To more rigorously assess the intervention’s efficacy, future research should conduct randomized control trials with larger and more diverse samples, while employing methods to improve assessment completion rates.

Additionally, the generalizability of the study is limited by the low demographic variability and small sample size. There were limitations with the generalizability of the study based on limited demographic differences within the sample. Due to these sample constraints, it was not possible to conduct analysis based on demographic data. The sample consisted primarily of Latine participants, which raises concerns about the intervention’s suitability for non-Latine undocumented young adults. Future studies should ensure that the demographics of participants are more representative of the undocumented young adult population. Most participants were also women, which aligns with previous research showing that women are more likely to seek out mental health support [66]. While this may reflect real-world help-seeking patterns, it is important to consider that men have historically experienced higher rates of detention and deportation [67]. Future research should make intentional efforts to recruit more men to better assess the efficacy of interventions with this population. Future research is also needed to confirm our preliminary findings regarding the effectiveness of using a randomized controlled trial design. Given the current immigration enforcement context and its impact on immigrant communities, it is recommended that this trial be implemented online with a waitlist control that receives access to the intervention after the study concludes. This will prioritize participant safety and reduce attrition rates that may be affected by risk associated with engaging in person mental health services during a period of heightened immigration enforcement.

Accessibility of the intervention is another key consideration. All participants had at least a high school diploma or General Education Development credential, and most had at least some college experience. This raises questions about the intervention’s accessibility for undocumented individuals with lower levels of formal education. Future versions of the intervention could benefit from feedback from community members across a broader range of educational experiences to ensure the language and content are widely accessible. In addition, most of the intervention was only available through written text. Feedback from participants suggested that audio versions of the intervention may help increase accessibility and engagement.

## Conclusions

Overall, the ACT Guide for Immigrant Resilience appears to be a promising intervention that could serve as a low-risk and accessible resource that could support the well-being of undocumented young adults. This intervention could be particularly useful as a prevention measure, a resource for individuals considering therapy, as a resource for those on a waitlist for therapy services, or a supplement for ongoing therapy. While preliminary results are promising, prospective randomized controlled trials are needed to confirm the effectiveness of this intervention in addressing mental health problems among undocumented young adults.

## Supporting information

S1 Fig Kendall’s Tau bivariate correlations between baseline measures and sessions/assessments completed. *** *p* < . 001, ** *p* < .01, * *p* < .05, no stars = *p* > . 05.
(TIFF)

S2 Fig Reliable change for statistically significant effects from pre-mid. (TIFF)

S3 Fig Reliable change for statistically significant effects from pre-post. (TIFF)

S4 Fig Reliable change for statistically significant effects from pre-follow-up. (TIFF)

S1 Table Outcome measures by timepoint. (DOCX)

S2 Table Participants with at least one missing variable for each timepoint by baseline scores. (DOCX)

S3 Table Codes with number of times they appeared. (DOCX)
