# Supplementary figures and images for: A pilot study of the online Acceptance and Commitment Therapy Guide for Immigrant Resilience: A culturally adapted intervention for undocumented community members
Source: PLOS Digit Health. 2026 Apr 3;5(4):e0001341. doi: 10.1371/journal.pdig.0001341 (PMC13048405; doi:10.1371/journal.pdig.0001341)

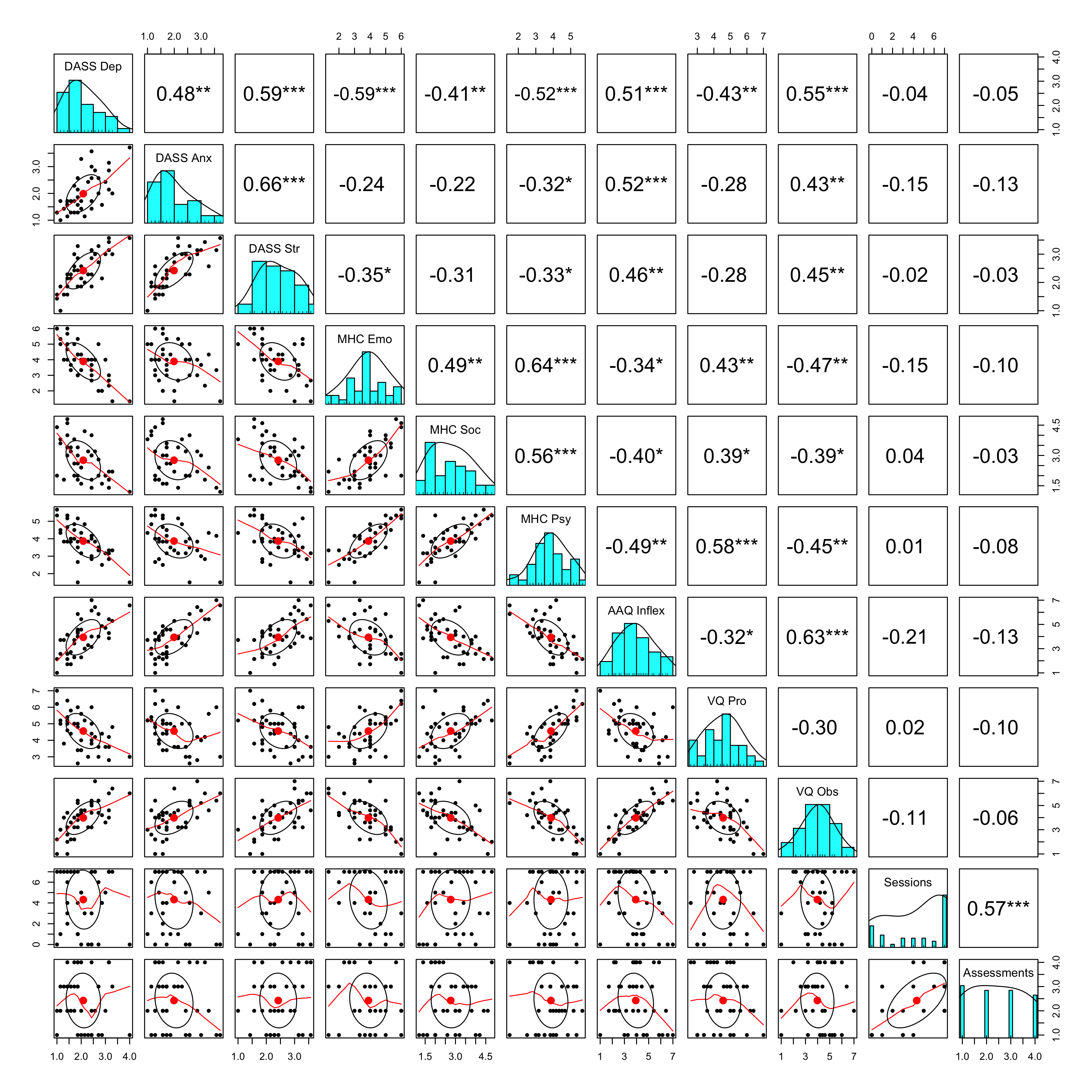

Supplement: S1 Fig — *** p < . 001, ** p < .01, * p < .05, no stars = p > . 05. (TIFF) [file pdig.0001341.s001.tiff]

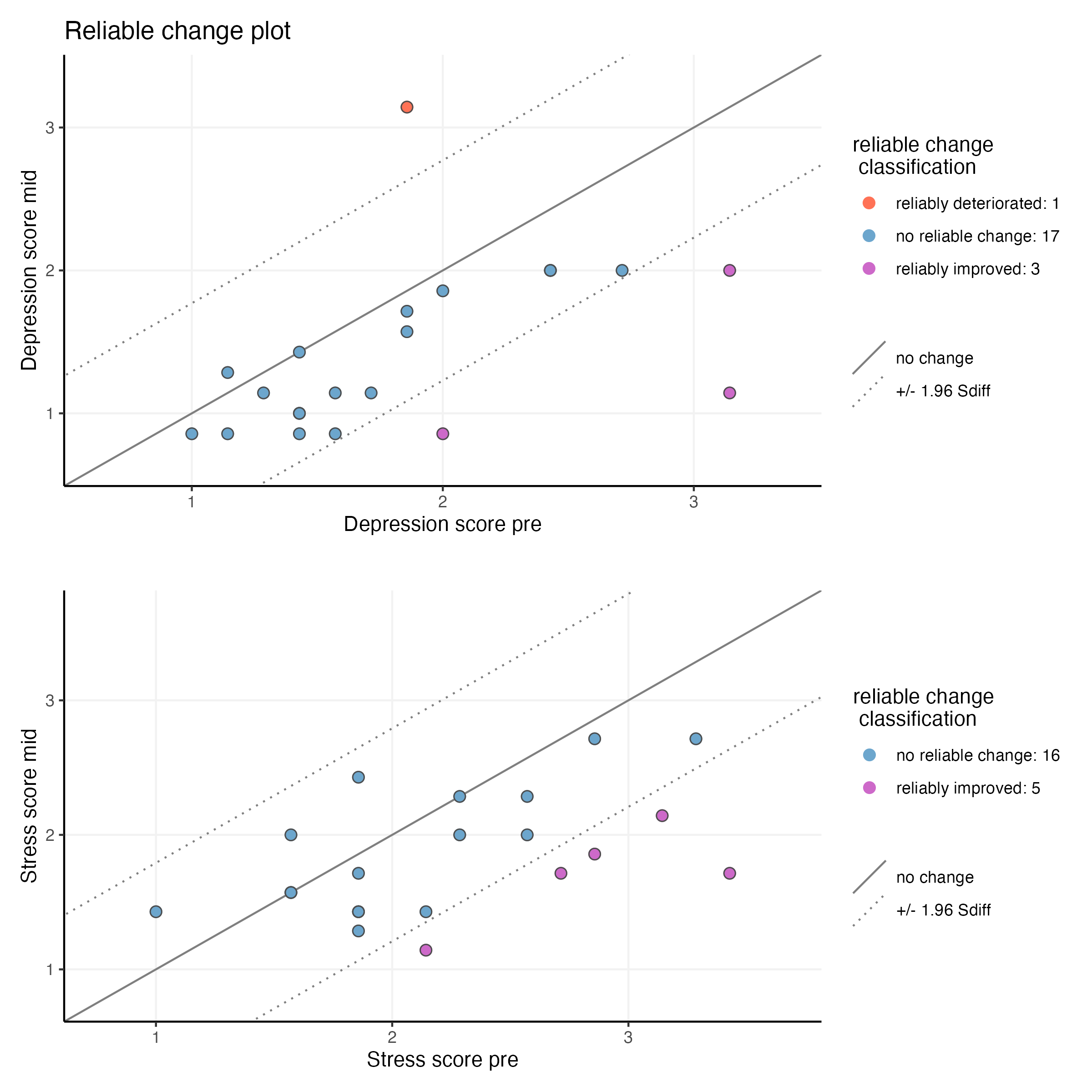

Supplement: S2 Fig — (TIFF) [file pdig.0001341.s002.tiff]

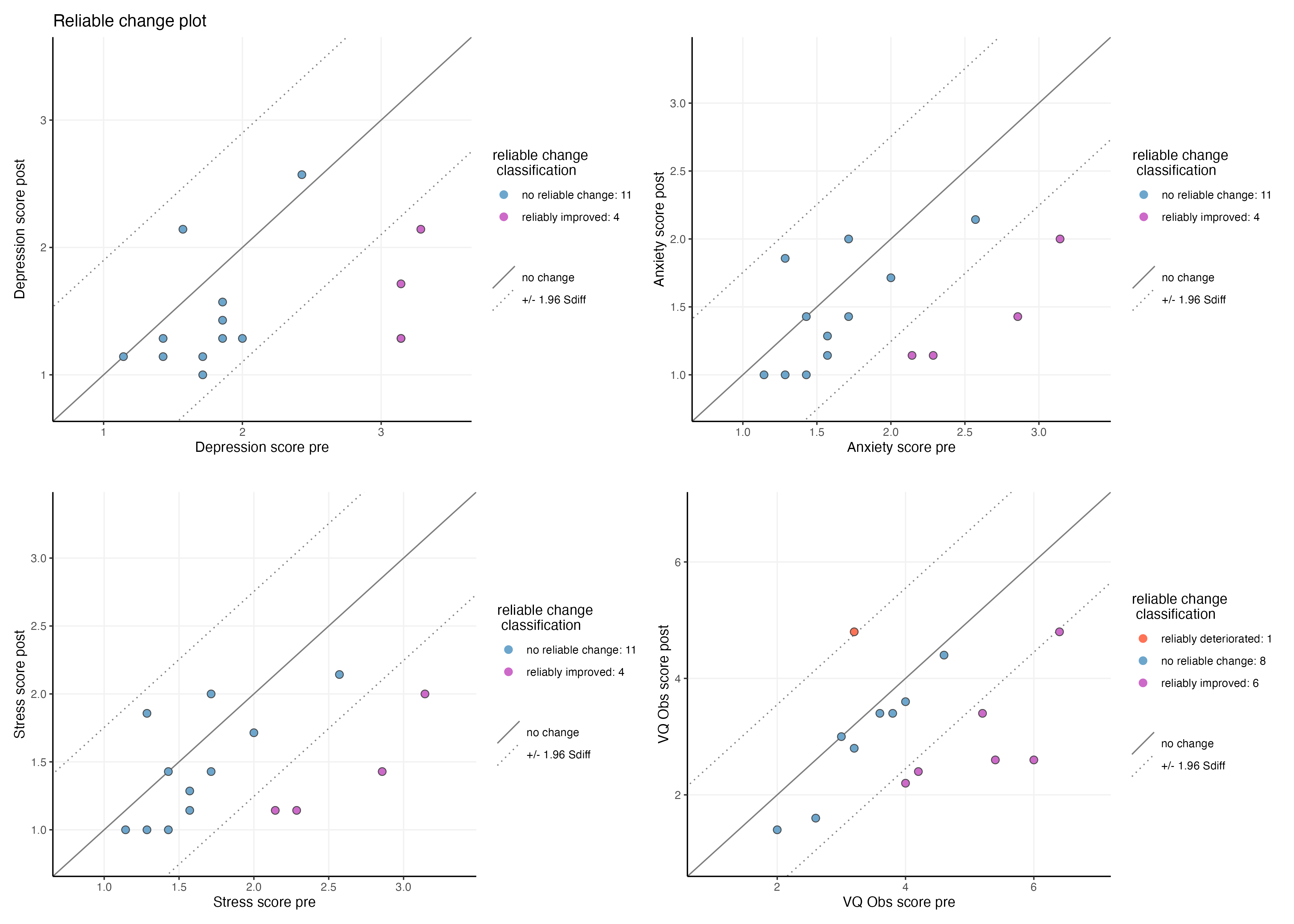

Supplement: S3 Fig — (TIFF) [file pdig.0001341.s003.tiff]

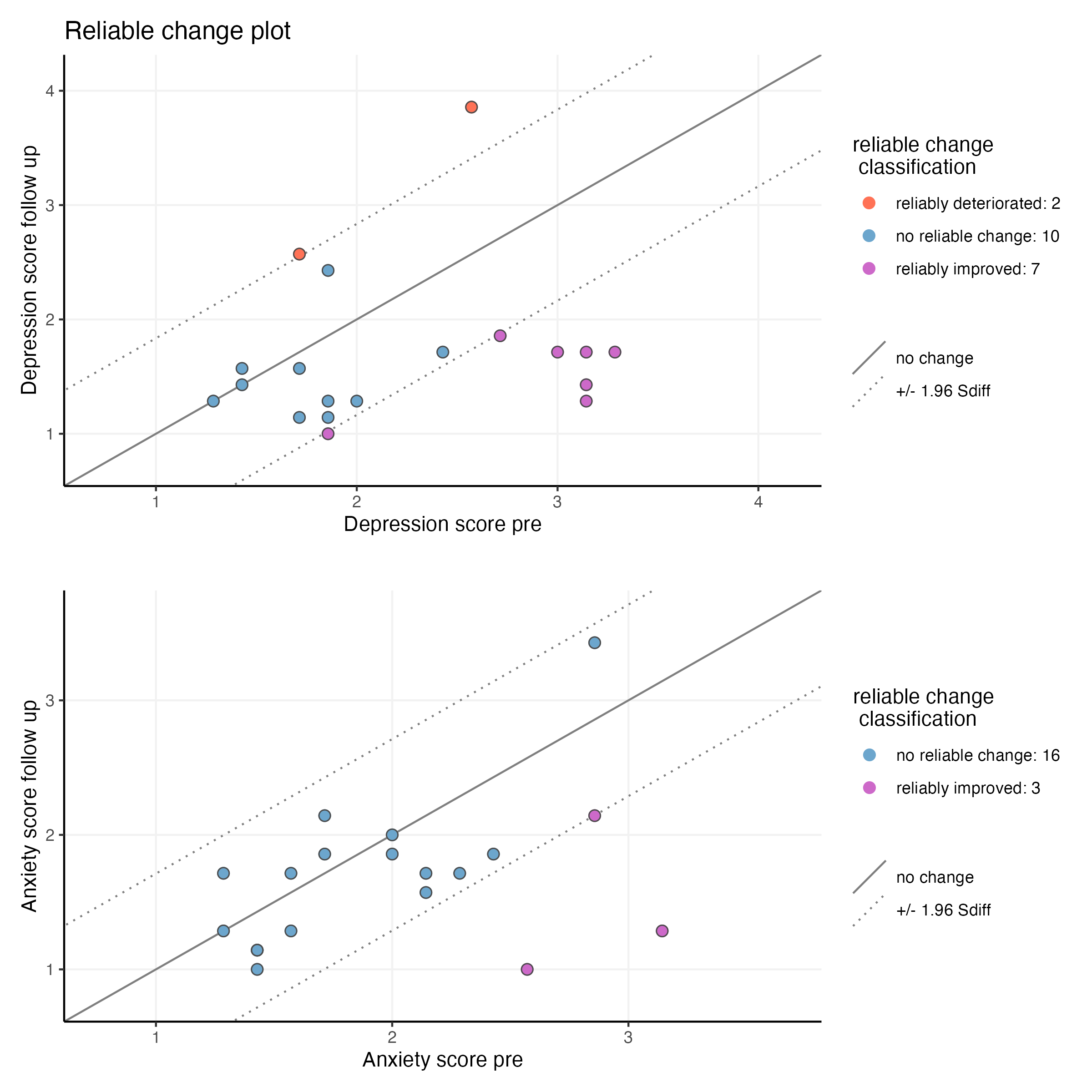

Supplement: S4 Fig — (TIFF) [file pdig.0001341.s004.tiff]
