# Supplementary material for: A pilot study of the online Acceptance and Commitment Therapy Guide for Immigrant Resilience: A culturally adapted intervention for undocumented community members
Source: PLOS Digit Health. 2026 Apr 3;5(4):e0001341. doi: 10.1371/journal.pdig.0001341 (PMC13048405; doi:10.1371/journal.pdig.0001341)
Supplement: S1 Table — (DOCX) [file pdig.0001341.s005.docx]

| **S1 Table**. **Outcome measures by timepoint.** | | | | | | | | |
| --- | --- | --- | --- | --- | --- | --- | --- | --- |
|  | ***n*** | ***M*** | ***SD*** | **Median** | **Min** | **Max** | **Skew** | **Kurtosis** |
| **Baseline** |  |  |  |  |  |  |  |  |
| DASS Dep | 40 | 2.09 | 0.71 | 1.93 | 1.00 | 4.00 | 0.53 | -0.39 |
| DASS Anx | 40 | 1.99 | 0.71 | 1.71 | 1.00 | 3.71 | 0.76 | -0.46 |
| DASS Str | 40 | 2.42 | 0.65 | 2.29 | 1.00 | 3.57 | 0.03 | -1.00 |
| MHC Emo | 40 | 3.89 | 1.21 | 4.00 | 1.33 | 6.00 | -0.17 | -0.53 |
| MHC Soc | 40 | 2.77 | 0.97 | 2.70 | 1.20 | 4.80 | 0.31 | -1.02 |
| MHC Psy | 40 | 3.87 | 0.97 | 3.83 | 1.50 | 5.67 | -0.34 | -0.10 |
| AAQ Inflex | 40 | 3.94 | 1.46 | 4.00 | 1.00 | 7.00 | 0.16 | -0.76 |
| VQ Pro | 40 | 4.56 | 1.06 | 4.70 | 2.60 | 7.00 | 0.10 | -0.71 |
| VQ Obs | 40 | 3.98 | 1.37 | 4.10 | 1.00 | 7.00 | -0.12 | -0.33 |
| **Mid** |  |  |  |  |  |  |  |  |
| DASS Dep | 23 | 1.53 | 0.68 | 1.29 | 0.86 | 3.14 | 0.99 | 0.14 |
| DASS Anx | 23 | 1.57 | 0.43 | 1.57 | 1.00 | 2.43 | 0.43 | -1.09 |
| DASS Str | 23 | 1.96 | 0.57 | 1.86 | 1.14 | 3.14 | 0.63 | -0.68 |
| MHC Emo | 22 | 4.03 | 1.19 | 4.17 | 2.00 | 6.00 | -0.18 | -1.19 |
| MHC Soc | 22 | 3.03 | 1.04 | 2.80 | 1.40 | 5.60 | 0.65 | -0.20 |
| MHC Psy | 22 | 3.64 | 1.08 | 3.42 | 2.00 | 5.50 | 0.28 | -1.37 |
| AAQ Inflex | 22 | 3.45 | 1.13 | 3.50 | 1.43 | 5.43 | -0.04 | -1.32 |
| VQ Pro | 21 | 4.72 | 0.94 | 4.60 | 2.80 | 6.60 | -0.05 | -0.39 |
| VQ Obs | 21 | 3.24 | 0.82 | 3.20 | 2.00 | 4.60 | -0.16 | -1.29 |
| **Post** |  |  |  |  |  |  |  |  |
| DASS Dep | 15 | 1.50 | 0.45 | 1.29 | 1.00 | 2.57 | 1.07 | -0.17 |
| DASS Anx | 15 | 1.45 | 0.40 | 1.43 | 1.00 | 2.14 | 0.42 | -1.44 |
| DASS Str | 15 | 1.79 | 0.38 | 1.71 | 1.29 | 2.43 | 0.13 | -1.56 |
| MHC Emo | 15 | 4.29 | 1.03 | 4.00 | 2.67 | 6.00 | 0.32 | -1.11 |
| MHC Soc | 15 | 3.57 | 1.31 | 3.40 | 1.60 | 6.00 | 0.23 | -1.01 |
| MHC Psy | 15 | 4.02 | 1.14 | 4.17 | 2.00 | 5.83 | -0.09 | -1.24 |
| AAQ Inflex | 15 | 3.01 | 0.92 | 3.14 | 1.57 | 4.43 | 0.13 | -1.26 |
| VQ Pro | 15 | 4.88 | 0.94 | 4.80 | 3.60 | 6.60 | 0.20 | -1.42 |
| VQ Obs | 15 | 3.09 | 1.04 | 3.00 | 1.40 | 4.80 | 0.17 | -1.02 |
| **Follow Up** |  |  |  |  |  |  |  |  |
| DASS Dep | 19 | 1.68 | 0.66 | 1.57 | 1.00 | 3.86 | 1.88 | 3.41 |
| DASS Anx | 19 | 1.68 | 0.56 | 1.71 | 1.00 | 3.43 | 1.35 | 2.41 |
| DASS Str | 19 | 2.04 | 0.64 | 1.86 | 1.14 | 3.43 | 0.67 | -0.80 |
| MHC Emo | 19 | 4.11 | 0.87 | 4.00 | 2.67 | 5.67 | 0.10 | -0.79 |
| MHC Soc | 19 | 3.18 | 1.21 | 3.40 | 1.60 | 6.00 | 0.49 | -0.68 |
| MHC Psy | 19 | 3.87 | 0.91 | 3.83 | 2.33 | 5.83 | 0.10 | -0.73 |
| AAQ Inflex | 19 | 3.23 | 1.38 | 2.71 | 1.14 | 6.71 | 0.74 | -0.15 |
| VQ Pro | 19 | 4.68 | 1.04 | 4.80 | 2.80 | 6.20 | -0.39 | -0.99 |
| VQ Obs | 19 | 3.43 | 1.40 | 3.20 | 1.60 | 7.00 | 0.82 | -0.01 |
| M = Mean, SD = standard deviation. DASS = Depression/Anxiety/Stress Scale; MHC Emo= Mental Health Continuum - Emotional; MHC Soc = Mental Health Continuum - Social; MHC Psy = Mental Health Continuum - Psychological; AAQ Inflex = Acceptance and Action Questionnaire ; VQ Pro = Valuing Questionnaire - Progress; VQ Obs = Valuing Questionnaire - Obstruction. | | | | | | | | |
