# Supplementary material for: A pilot study of the online Acceptance and Commitment Therapy Guide for Immigrant Resilience: A culturally adapted intervention for undocumented community members
Source: PLOS Digit Health. 2026 Apr 3;5(4):e0001341. doi: 10.1371/journal.pdig.0001341 (PMC13048405; doi:10.1371/journal.pdig.0001341)
Supplement: S2 Table — (DOCX) [file pdig.0001341.s006.docx]

| **S2 Table**. **Participants with at least one missing variable for each timepoint by baseline scores.** | | | | | | | | | |
| --- | --- | --- | --- | --- | --- | --- | --- | --- | --- |
|  | **Mid** | |  | **Post** | |  | **Follow Up** | |  |
|  | **Incomplete** | **Complete** | **p-value** | **Incomplete** | **Complete** | **p-value** | **Incomplete** | **Complete** | **p-value** |
|  | *n* = 17 | *n* = 23 ^a^ |  | *n* = 25 | *n* = 15 |  | *n* = 21 | *n* = 19 |  |
| DASS Dep | 2.37 (0.70) | 1.88 (0.66) | .03 | 2.07 (0.72) | 2.11 (0.72) |  | 1.97 (0.74) | 2.22 (0.67) |  |
| DASS Anx | 2.25 (0.75) | 1.80 (0.63) | .04 | 2.06 (0.77) | 1.88 (0.61) |  | 1.99 (0.83) | 1.99 (0.57) |  |
| DASS Str | 2.60 (0.59) | 2.29 (0.67) |  | 2.42 (0.65) | 2.43 (0.68) |  | 2.29 (0.64) | 2.57 (0.65) |  |
| MHC Emo | 3.75 (1.23) | 4.00 (1.20) |  | 4.03 (1.19) | 3.67 (1.24) |  | 4.16 (1.24) | 3.60 (1.13) |  |
| MHC Soc | 2.56 (0.92) | 2.91 (1.00) |  | 2.82 (0.96) | 2.68 (1.01) |  | 2.96 (1.03) | 2.55 (0.87) |  |
| MHC Psy | 3.70 (0.93) | 3.99 (1.01) |  | 3.97 (0.93) | 3.69 (1.05) |  | 4.06 (1.00) | 3.65 (0.92) |  |
| AAQ Inflex | 4.55 (1.62) | 3.48 (1.18) | .02 | 4.02 (1.63) | 3.79 (1.18) |  | 3.88 (1.65) | 3.99 (1.27) |  |
| VQ Pro | 4.41 (1.13) | 4.67 (1.02) |  | 4.68 (1.12) | 4.36 (0.96) |  | 4.88 (1.14) | 4.21 (0.87) |  |
| VQ Obs | 4.40 (1.51) | 3.67 (1.20) |  | 3.92 (1.46) | 4.08 (1.25) |  | 3.79 (1.41) | 4.19 (1.33) |  |
| Three participants were excluded who provided partial information on study scales. Incomplete = participants missing at least one variable. DASS = Depression/Anxiety/Stress Scale; MHC Emo= Mental Health Continuum - Emotional; MHC Soc = Mental Health Continuum - Social; MHC Psy = Mental Health Continuum - Psychological; AAQ Inflex = Acceptance and Action Questionnaire ; VQ Pro = Valuing Questionnaire - Progress; VQ Obs = Valuing Questionnaire - Obstruction. | | | | | | | | | |
|  |  |  |  |  |  |  |  |  |  |
