# Supplementary material for: A pilot study of the online Acceptance and Commitment Therapy Guide for Immigrant Resilience: A culturally adapted intervention for undocumented community members
Source: PLOS Digit Health. 2026 Apr 3;5(4):e0001341. doi: 10.1371/journal.pdig.0001341 (PMC13048405; doi:10.1371/journal.pdig.0001341)
Supplement: S3 Table — (DOCX) [file pdig.0001341.s007.docx]

**S3 Table**. **Codes with number of times they appeared.**

| Session | Liked best | Learned | Liked least |
| --- | --- | --- | --- |
| 1 | Relatable content (13)  Self-compassion (12)  Self-reflection (6)  Easy navigation (2) | Self-compassion (12)  Self-credit (10)  Self-reflection (6)  Not alone (3) | No dislikes (20)  Emotionally taxing (3)  Too much info (3)  Too long (3)  Request for auditory option (2) |
| 2 | Values reflection (18)  Insight on values vs rules (9)  Examples (3)  Relatable content (2) | Importance of values (14)  Values vs rules (10)  Self-compassion (3) | No dislikes (21)  Unclear section (2)  Content suggestion (2) |
| 3 | Reflection on avoidance (7)  Metaphors (10)  Examples (5)  Self-reflection (2)  Skills (2)  Explanation of away moves (2) | Consequences of avoidance/away moves (17)  Identifying away moves (4)  Openness to feelings (2)  Expressing emotions (2) | No dislikes (16)  Confusing (4)  Request for substitution to away moves (3) |
| 4 | Concept of leaning back (7)  Leaning back (activities) (8)  Self-reflection (3)  Assignment (2)  Not applicable (2)  Simplified explanation (2) | Leaning back (18)  Perspective (8)  Acceptance (2) | No dislikes (14)  More examples (3)  Short (3)  More detail (2) |
| 5 | Guided mindfulness (16)  Tinted glasses metaphor (4)  Activities (2) | Defusion (11)  Mindfulness practice (7)  Perspective (2) | No dislikes (12)  Audio challenges (3)  More guided exercises (2)  Too many exercises (2) |
| 6 | Noticing 'buts' (5)  Taking control (4)  Passengers on the bus metaphor (4)  Examples (3)  Acceptance (2)  tools for navigating emotions (2) | Coexisting with feelings (11)  Doing what matters (4)  Changing 'buts' to 'and' (3) | No dislikes (11)  More clarity (3)  Needs audios/videos (2) |
| 7 | Reflection on values and actions (9)  Letter to self (5)  Resources (2)  90th birthday exercise (2)  Adverb activity (2) | Reflection on values and actions (11)  Taking control (3)  Using adverbs (2)  Self-compassion (2) | No dislikes (10)  Simplify (3) |
